# Role of the LuxR solo, SdiA, in eavesdropping on foreign bacteria

**DOI:** 10.1093/femsre/fuaf015

**Published:** 2025-04-16

**Authors:** Andrew Schwieters, Brian M M Ahmer

**Affiliations:** Department of Microbiology, The Ohio State University, Columbus, OH 43210, United States; Department of Microbiology, The Ohio State University, Columbus, OH 43210, United States; Department of Microbial Infection and Immunity, The Ohio State University, Columbus, OH 43210, United States

**Keywords:** quorum sensing, SdiA, eavesdropping, acyl homoserine lactones, *Salmonella*, *E. coli*

## Abstract

Bacteria can cooperate by coordinating their gene expression through the production, release, and detection of small molecules, a phenomenon known as quorum sensing (QS). One type of QS commonly found in Gram-negative bacteria utilizes a LuxI-type enzyme to produce a signaling molecule of the *N*-acyl-homoserine lactone (AHL) family, and a transcription factor of the LuxR family to detect and respond to the AHL. In a subset of Enterobacteriaceae, including *Escherichia coli* and *Salmonella*, no LuxI family member is present and no AHLs are synthesized. However, they encode a LuxR family member, SdiA, that is used to detect the QS molecules of other bacterial species, a behavior known as eavesdropping. Despite significant research on the topic, the overall role of SdiA-mediated eavesdropping in these bacteria remains unclear. In this review, we discuss the phenotypes and regulons of SdiA in the Enterobacteriaceae.

## Teaser

A review of the current literature on LuxR solo, SdiA, an eavesdropping protein found in a subset of the family Enterobacteriaceae.

## Introduction

Quorum sensing (QS) is a behavior that allows bacteria to measure their population density by producing and releasing small molecules into the surrounding environment. In suitable environments (e.g. sufficient population density and areas of low diffusion), these small molecules reach a detectable threshold concentration, leading to coordinated behaviors in the population through a ligand bound response regulator. QS itself is a highly reviewed topic (Fuqua et al. 1994, Redfield 2002, Papenfort and Bassler 2016, Whiteley et al. 2017, Mukherjee and Bassler 2019, Aframian and Eldar 2020, Duddy and Bassler 2021). In this review, we refer only to QS in Gram-negative bacteria utilizing small molecules of the *N*-acyl-homoserine lactone (AHL) family unless otherwise stated. AHLs contain a homoserine lactone ring, amide group, and variable length acyl side group that can be modified by carbonyl or hydroxyl substitution on the third carbon. A QS circuit encodes an AHL synthase (LuxI or LuxM) and the response regulator (LuxR) that detects the AHL.

There is a growing appreciation in the field of QS for LuxR proteins that lack a cognate synthase, which have been broadly termed orphans or “LuxR solos” (Patankar and González 2009, Subramoni and Venturi 2009, Venturi and Ahmer 2015). These lone regulators can be classified by the source of their ligand (endogenous or exogenous) and the ligand itself (AHL, non-AHL, or none) (Bez et al. 2023). In some cases, LuxR solos may bind endogenously produced AHLs from another QS circuit on the genome (“third-wheels”). QscR is one of the better studied LuxR solos that acts as a third-wheel in *Pseudomonas aeruginosa*. QscR activity is regulated by binding *N*-(3-dodecanoyl)-homoserine lactone (oxoC12), an AHL produced by AHL synthase LasI of the LasRI QS circuit, encoded elsewhere on the *P. aeruginosa* genome (Chugani et al. 2001, Lequette et al. 2006, Ding et al. 2018). Interestingly, QscR can also bind AHLs other than those produced by *P. aeruginosa* (Oinuma and Greenberg 2011), suggesting it may also act as a detector of foreign bacteria, a behavior known as eavesdropping or alloinduction (Greenberg et al. 1979).

**Figure 1.**
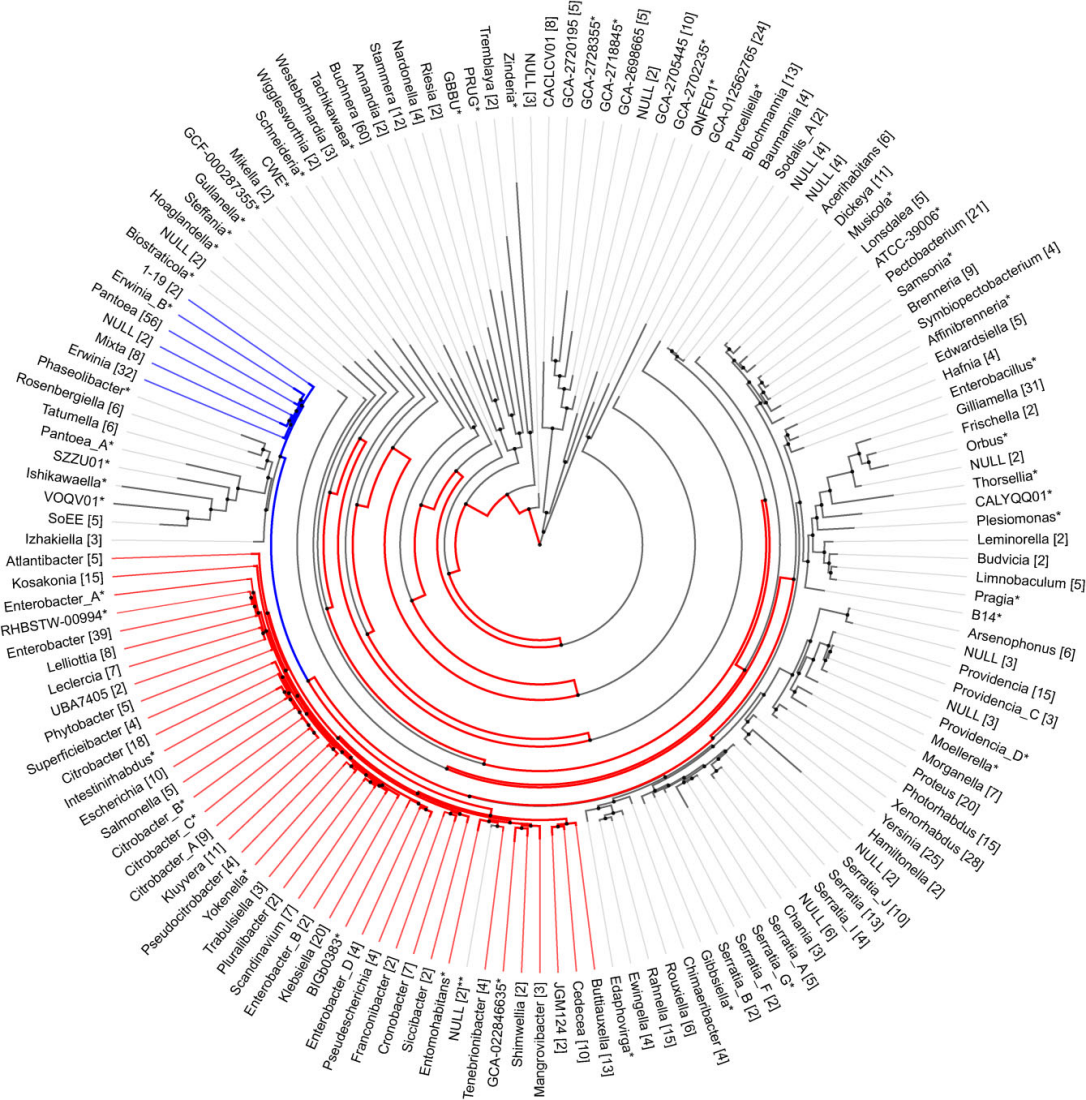
Bacterial genera predicted to undergo SdiA-mediated eavesdropping in the Enterobacteriaceae. Genomes within the family Enterobacteriaceae were searched with BLAST using *S. enterica* subspecies *enterica* serovar Typhimurium 14028 SdiA as an input. AnnoTree (Mendler et al. 2019) version 214 was used to identify taxonomies that encode *sdiA* based on the BLAST search (marked red). There were taxonomies with putative hits for *sdiA* outside of the region spanning *Atlantibacter* to *Buttiauxella*. These genomes were manually examined to determine whether *sdiA* was truly present in the correct genomic region (e.g. adjacent to *uvrY*). If it was not, it was manually colored black. If it was adjacent to *uvrY* and there was an AHL synthase, it was marked blue. Within the region spanning *Atlantibacter* to *Buttiauxella*, there were some taxonomies marked black as if they did not have *sdiA*. These were manually examined to determine if *sdiA* was truly present and adjacent to *uvrY*. If so, they were colored red. Numbers in brackets indicate number of genomes per taxonomic group. *only one genome in that taxonomic group. ** Genomes unavailable.

**Figure 2.**
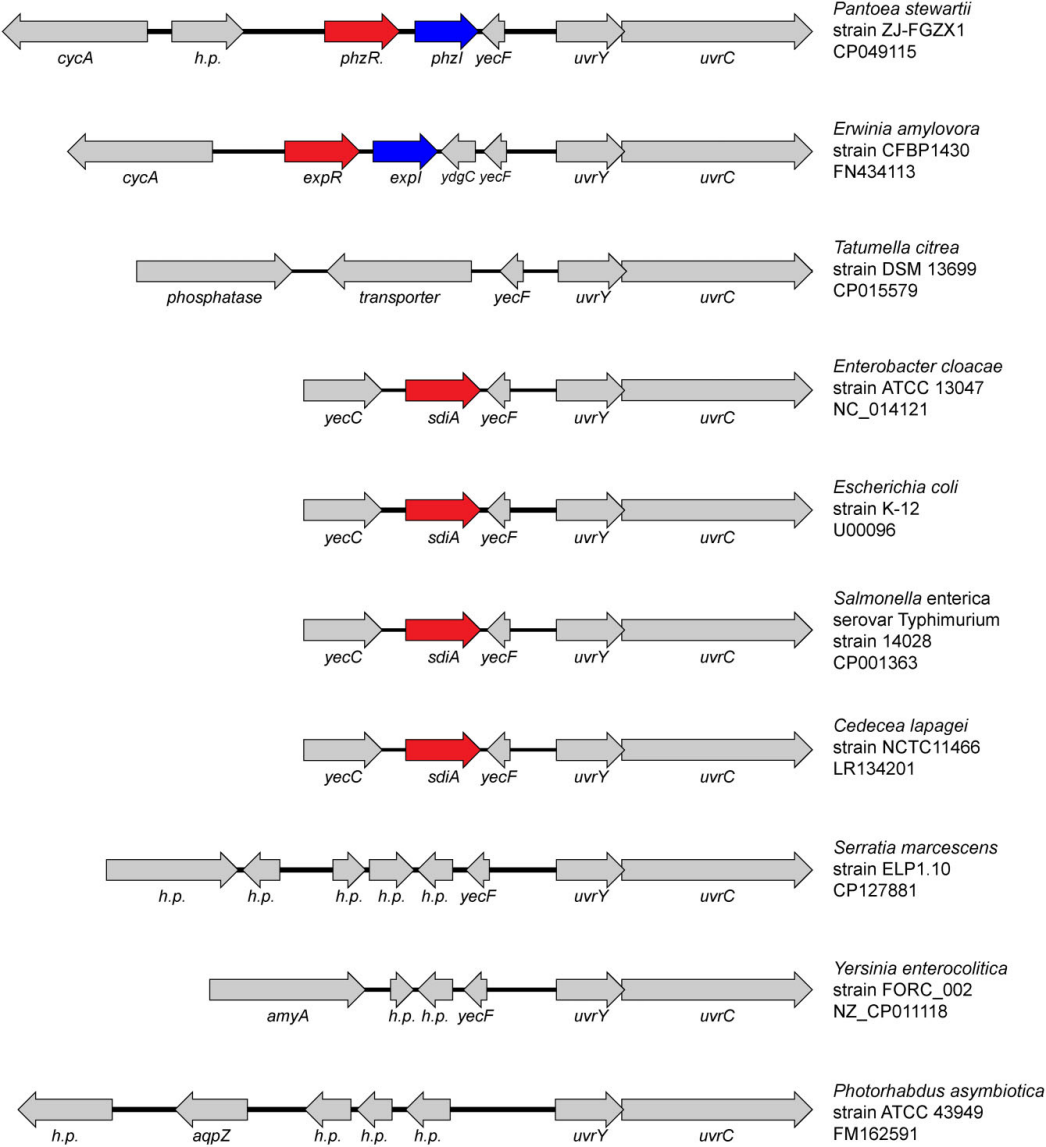
Genomic organization upstream of the *uvrYC* locus in the family Enterobacteriaceae.

The subject of this review is SdiA, a LuxR solo found in several bacterial genera within the family Enterobacteriaceae (Fig. 1) (Michael et al. 2001, Ahmer 2004). SdiA is one of the most studied LuxR solos mediating eavesdropping (Michael et al. 2001). The genomic context of *sdiA* always includes a gene downstream that encodes the transcription factor, UvrY. Genera including *Erwinia* and *Pantoea* encode a LuxI homolog in between *sdiA* and *uvrY*, suggesting that SdiA was once part of this ancestral LuxR/LuxI pair (Sabag-Daigle and Ahmer 2012) (Fig. 2). The divergence of *Salmonella* and *Escherichia* has been estimated to have occurred between 60 and 100 million years ago, and the divergence of *Escherichia* and *Erwinia* is much older (Ochman and Wilson 1987, Ochman and Groisman 1994, Lawrence and Ochman 1998). Thus, the adoption of SdiA-mediated eavesdropping is a relatively ancient event. Despite such a long time frame, the presence of *sdiA* is maintained within these genera, even in recently emerged bacteria like *Salmonella enterica* serovar Typhi (Schwieters and Ahmer 2024). Therefore, the detection of AHLs produced by other bacteria is likely a valuable function.

While SdiA-mediated eavesdropping is easily demonstrated in laboratory environments (Smith and Ahmer 2003, Smith et al. 2008, Dyszel et al. 2010a, Noel et al. 2010), its role in the lifecycle of bacteria in nature remains elusive. This is due in part to the absence of clear-cut phenotypes of *sdiA* mutants, but also the complex, sometimes contradictory collection of studies that require more nuanced consideration. In this review, we discuss the current body of literature on SdiA, focusing on its reported phenotypes and regulons. Each section discusses an important yet incompletely answered question in the field.

## How is SdiA activity regulated by AHLs?

SdiA is a LuxR-type protein, a family named for the response regulator which controls bioluminescence in *Vibrio fischeri* (Engebrecht et al. 1983, Engebrecht and Silverman 1984, Kaplan and Greenberg 1987). This is not to be confused with the LuxR protein of *Vibrio harveyi*, which contains a TetR-type helix-turn-helix domain (Pompeani et al. 2008). LuxR-type proteins are two domain proteins encoding a N-terminal ligand-binding domain and C-terminal helix-turn-helix domain responsible for binding to DNA by recognizing a specific motif, usually as a homodimer (heterodimers of two LuxR-type proteins have been reported in *P. aeruginosa*; Ledgham et al. 2003). Depending on the specific protein, transcriptional activation can occur by both class I and class II mechanisms while repression has been shown to occur through steric hindrance (Egland and Greenberg 1999, White and Winans 2005, Castang et al. 2006). The regulatory mechanisms for SdiA activation and repression have not been experimentally determined, with the exception of one study suggesting a class II mechanism of *Escherichia coli* SdiA on the *ftsQAZ* promoter (Yamamoto et al. 2001). However, the relevance of the interaction between *E. coli* SdiA and the *ftsQAZ* promoter requires further consideration (see below).

Schuster and Greenberg (2008) proposed a classification scheme for LuxR-type proteins based on folding and ligand-binding characteristics. Class I proteins, such as TraR *of Agrobacterium tumefaciens*, require AHL for folding and bind them irreversibly (Zhu and Winans 2001). Class II proteins, such as *V. fischeri* LuxR, also require AHL for folding but bind them reversibly (Urbanowski et al. 2004). Class III proteins, such as *ExpR* of Erwinia (which is an SdiA ortholog), do not require AHLs for folding and bind them reversibly (Castang et al. 2006). *Escherichia coli* SdiA can be purified in the absence of AHL (possibly requiring an endogenous ligand, 1-octanoyl-*rac*-glycerol) suggesting it fits into class III (Kim et al. 2014, Nguyen et al. 2015). Regulation by endogenously produced ligands and non-AHL ligands, as well as regulation independent of ligands, have been described with other LuxR solos (Bez et al. 2023). Orthologs of SdiA in *Salmonella, E. coli*, and *E. cloacae* also have both AHL-dependent and -independent regulatory phenotypes, supporting the notion that AHLs are dispensable for folding (Dyszel et al. 2010b, Sabag-Daigle et al. 2015, Schwieters and Ahmer 2024). Apo-SdiA forms an open ligand-binding pocket that limits the size of the acyl chain through two residues, F59 and L77 (Nguyen et al. 2015). In *Salmonella*, these residues are flipped (L59 and F77). Both *Salmonella* and *E. coli* SdiA preferentially interact with mid chain length AHLs, specifically *N*-(3-oxohexanoyl)-homoserine lactone (oxoC6) and *N*-(3-oxooctanoyl)-homoserine lactone (oxoC8). At least for *Salmonella*, AHL-dependent SdiA activity can be induced by both shorter and longer chain lengths, suggesting the detectable range of foreign AHL producing bacteria is relatively broad (Michael et al. 2001, Panchal et al. 2024).

It is still unclear how AHLs lead to SdiA-dependent changes in transcriptional activity. The AHL bound state is only slightly different than apo-SdiA in structure (Nguyen et al. 2015). It is possible this small difference is sufficient for altering the binding motif as the *ler* promoter of *E. coli* O157::H7 contains two SdiA binding sites: one AHL-independent and one AHL-dependent (Nguyen et al. 2015). An SdiA box has been proposed based on DNase I footprinting of *E. coli* SdiA at the *ftsQAZ* promoter, but this site cannot be found in the *ler* promoter or upstream of *Salmonella* SdiA regulated genes (*Salmonella* and *E. coli* SdiA are 71% identical at the amino acid level) (Yamamoto et al. 2001, Abed et al. 2014). AHLs could also regulate through altering the stability of SdiA (Nguyen et al. 2015), but this is not consistent with the existence of some loci that are regulated by SdiA in the absence of AHL (Sabag-Daigle et al. 2015, Schwieters and Ahmer 2024). Adding to the confusion is the fact that AHL-dependent regulation at some promoters becomes partially AHL-independent at 30°C (compared to 37°C) in *E. coli* and *Salmonella* (Smith and Ahmer 2003, Dyszel et al. 2010b). The underlying mechanism of temperature dependent effects on regulation has not been investigated.

## Where and when is SdiA relevant *in vivo*?

Of considerable significance to understanding SdiA-mediated eavesdropping is identifying the relevant environment in which it occurs. Eavesdropping bacteria are found in diverse environmental niches, but experimental data primarily comes from infections of animal models using two intestinal pathogens: Enterohemorrhagic *E. coli* (EHEC) and *Salmonella enterica* serovar Typhimurium (*S*. Typhimurium). Below we describe the techniques used and the relevant studies for each environment evaluated.

### Methodology

There are three predominant methods for evaluating the relevance of an environment for SdiA-mediated gene regulation. The first method is to determine if *sdiA* provides a fitness advantage in a particular environment. This is often done using a competition assay, in which a 1:1 ratio of wild-type and *sdiA* mutant are inoculated into the system. Later, the bacteria can be sampled from the environment and their ratio measured again. Changes from the initial ratio then indicate fitness phenotypes and biological relevance may be inferred. A second method is to determine if SdiA has become active while in a particular environment. We have used a reporter strain of *S*. Typhimurium that utilizes site-specific recombination to measure SdiA activity (Camilli et al. 1994, Slauch and Camilli 2000). This reporter will heritably delete an antibiotic resistance marker from its chromosome in the presence of AHLs in an *sdiA*-dependent manner (Smith et al. 2008, Dyszel et al. 2010a, Noel et al. 2010). By inoculating environments with both wild-type and *sdiA* mutant reporter strains of *S*. Typhimurium, fitness contributions and SdiA activity can be evaluated simultaneously. A third method to assess the relevance of an environment is to look for AHLs (i.e. chemical detection) or the abundance of AHL-producing bacteria.

In Table 1, we show the results of a search for LuxI homologs in the IMG database, where any metagenome containing at least one predicted LuxI homolog is marked positive. This analysis is insufficient to make conclusions about the abundance of AHLs in an environment or the number of AHL-producing bacteria in a community. However, the analysis is sufficient to identify those microbiomes that do not include *luxI* homologs. For example, >99% of human gut microbiomes do not include a *luxI* homolog. These results and their implications are discussed in their appropriate sections below.

**Table 1.** The frequency of *luxI* orthologs in metagenomes. Predicted proteins of metagenomes of the indicated hosts were searched for homologs of AHL synthase *luxI* (*V. fischeri*) using the IMG blastp search, with a minimum bit score of 60. Any metagenome with at least one hit was considered positive.

Host	Total number of metagenomes	Number of metagenomes with 1 or more LuxI homologs	Percent of metagenomes with 1 or more LuxI homologs (%)
Annelida	149	33	22.1
Arthropoda (digestive)	124	21	17.0
Arthropoda (other)	231	67	29.0
Birds	38	6	15.8
Fish	11	6	54.4
Human (digestive)	2364	20	0.8
Human (skin)	674	121	18.0
Human (other)	356	4	1.1
Nonhuman mammal (digestive)	734	24	3.3
Nonhuman mammal (other)	21	8	38.0
Plants (phyllosphere)	572	138	24.1
Plants (rhizome)	180	138	76.7
Plants (roots)	630	392	62.2
Plants (other)	62	20	32.2

### Mice and humans

The plant-associated genera, *Erwinia* and *Pantoea*, encode a *luxI* homolog adjacent to their SdiA ortholog (Fig. 2). In the subset of Enterobacteriaceae where this *luxI* gene has been deleted, these genera are largely associated with the gastrointestinal tract. Thus, one hypothesis is that the *luxI* homolog was lost during evolution because there is no need to synthesize AHLs in an AHL-replete environment such as the gastrointestinal tract. However, the gastrointestinal tract does not appear to have a relevant concentration of AHLs. Metagenomic data suggests a lack of AHL producing microbiota in the mouse gut (Smith et al. 2008, Dyszel et al. 2010a, Swearingen et al. 2013), and indeed very few of the searched human and mammalian gut microbiomes encode one or more *luxI* homologs (Table 1). Additionally, an *S*. Typhimurium reporter of SdiA activity is not active during transit through the gastrointestinal tract of mice nor do *sdiA* mutants exhibit significant fitness defects in mice (Smith et al. 2008, Dyszel et al. 2010a). Therefore, it appears that SdiA is not utilized for detecting AHLs produced by the normal microbiota of the gastrointestinal tract of mice.

However, some gastrointestinal pathogens do synthesize AHLs. Could the purpose of SdiA be to detect coinfections with AHL-producing pathogens? If mice are first infected with the AHL-producing pathogen *Yersinia enterocolitica*, SdiA of *S*. Typhimurium does indeed become active during transit through these animals (Dyszel et al. 2010a). However, the *sdiA* mutant has no fitness defect in this scenario. It is possible that *Y. enterocolitica* is not a relevant AHL-producing pathogen for *Salmonella. Yersinia* and *Salmonella* may not interact sufficiently or in the correct manner to invoke a fitness contribution from SdiA. To test the contribution of AHL availability to *Salmonella* fitness more directly, the *yenI* gene (encoding the AHL synthase YenI) was placed into the chromosome of *Salmonella*, enabling *Salmonella* to produce AHLs without the need for another bacteria (Dyszel et al. 2010a). In this strain background, the *sdiA* mutant is attenuated in the mouse gut during infection and it is the largest *sdiA* mutant defect observed to date (>100-fold) (Dyszel et al. 2010a). The fitness advantage conferred by *sdiA* requires its regulon members, *pefI-srgC* and *srgE* (Dyszel et al. 2010a). Therefore, it appears that the SdiA regulon could provide a beneficial function in the gut if AHLs were to be encountered. This is paradoxical given the lack of AHLs, or AHL-producing organisms, in gut environments. Such results raise an interesting question as to why *Escherichia, Salmonella*, and other SdiA-encoding genera wait for an AHL signal to be provided to them to express these beneficial genes. SdiA regulated genes may in fact be beneficial only when those AHLs reach detectable concentrations.

Humans cannot be tested directly, but the possibility of QS in the human gut was recently reviewed (Oliveira et al. 2023). Bioinformatic searches find almost no *luxI* homologs in the human gut microbiome (Table 1; Swearingen et al. 2013, Grellier et al. 2022), yet AHLs have been directly detected in both the gut and feces (Landman et al. 2018, Xue et al. 2021, Grellier et al. 2022). These direct measurements find that AHL concentrations are in the low nanomolar range, near the detection limit of SdiA (Michael et al. 2001, Styles and Blackwell 2018), but this could be higher in microenvironments (Mukherjee and Bassler 2019). QS potential is further complicated by antagonistic compounds in the gut (e.g. indole), quorum quenching activity (e.g. lactonases), and shifts in microbiome composition shifts during infection (Sabag-Daigle et al. 2012, Stecher et al. 2013, Grandclément et al. 2015, Borton et al. 2017, Argüello et al. 2018, Rogers et al. 2021). The relevance of QS in the human/mouse gut to eavesdropping bacteria like *Salmonella* remains an open question.

### Cattle

Cattle have been explored as a site of SdiA activity using *S*. Typhimurium and *E. coli* O157:H7. The *Salmonella* reporter system described above has only been tested in a single calf, but there was no activation of SdiA or *sdiA* mutant defect (Smith et al. 2008). *Escherichia coli* has been tested in larger cohorts using competition assays and single infections that indicate fecal shedding and colonization defects to varying degrees (up to ∼4-fold) (Hughes et al. 2010, Sperandio 2010, Sharma and Bearson 2013, Sheng et al. 2013). AHLs have also been extracted from the rumen with seasonal and dietary effects on concentration (Hughes et al. 2010, Sperandio 2010, Sheng et al. 2013). The intensity of *sdiA* mutant defects positively correlates with diets that increase AHL concentration in the rumen. From these studies, a model has been proposed whereby pathogenic *E. coli* sense AHLs in the rumen to activate their acid response system and suppress virulence (Edrington et al. 2009, Hughes et al. 2010, Sperandio 2010). SdiA-mediated suppression of virulence is alleviated upon leaving the rumen, allowing for colonization of the gastrointestinal tract. However, both acid tolerance and virulence have a degree of AHL-independent regulation by SdiA and the rumen microbiota member(s) producing AHLs have yet to be identified.

### Plants

Plant microbiomes are known to include AHL-producing pathogens and commensals (González and Marketon 2003, von Bodman et al. 2003, Hartmann et al. 2014, Cellini et al. 2020). They also showed a high frequency of detection of *luxI* homologs in our pilot metagenome search (Table 1). One of the interesting but largely overlooked aspects of SdiA-mediated eavesdropping is that *Erwinia* and *Pantoea*, two genera still encoding the ancestral cognate AHL synthase of SdiA, are known plant colonizers (Starr and Chatterjee 1972, Walterson and Stavrinides 2015). Additionally, many of the currently known LuxR solos are encoded in plant colonizing bacteria (Bez et al. 2023). Based on these circumstantial factors, the hypothetical relationship between SdiA and survival in, or near, plants is the strongest current hypothesis. Unfortunately, only two studies have evaluated this idea *in vivo* and they are insufficient to draw conclusions (Noel et al. 2010, Shankar et al. 2013). The experimental data on this putative relationship is described below.

Plants have been probed for *Salmonella* SdiA activation in tomato soft rot caused by the plant pathogen *Pectobacterium carotovorum* (Noel et al. 2010). Although *P. carotovorum* produces AHLs detectable by *Salmonella in vitro*, there is no SdiA activity during coinfection within the plant (and *sdiA* mutants have no fitness defects) (Noel et al. 2010). The lack of detection was attributed to lack of transcription of *sdiA* (Noel et al. 2010). The transcription of *Salmonella sdiA* is primarily regulated by FliA, Crp, and LeuO (Turnbull et al. 2011, Plitnick et al. 2020) and it is unknown if this loss of activation occurs through modulating expression of these upstream regulators. A second study on a possible plant–SdiA relationship found that rice root extracts have detectable concentrations of AHLs (via biosensor strains) and *sdiA* mutation enhances root colonization of *E. cloacae* (Shankar et al. 2013). The advantage conferred by *sdiA* mutation suggests that plants may not be a relevant environment, at least for *E. cloacae*. In a small pilot study, our lab inoculated the soil and leaves of a variety of commercially available Angiosperms (leeks, parsley, tomato, and soybeans) with our *Salmonella* reporter strains but found no activation of SdiA or fitness defects of the *sdiA* mutant (unpublished data).

### Insects

Insects are known reservoirs and transmission vectors of genera that encode *sdiA*, such as *Salmonella* (Wales et al. 2010, Blazar et al. 2011), and their microbiota are commonly colonized with AHL producing genera like *Pseudomonas* and *Pantoea* (Engel and Moran 2013). Based on our pilot exploration of IMG metagenomes, *luxI* homologs are detected in arthropod metagenomes more frequently than in human and nonhuman mammalian samples (Table 1). Therefore, one could hypothesize that insect guts are a relevant environment that include AHL-producing bacteria detected by SdiA. Like plants, little data has been collected to support this possibility. AHL producing *Rahnella* species have been isolated from the gut of wax moth larvae (*Galleria mellonella*) (de Freitas et al. 2022). Subsequent infections of *G. mellonella* with *Salmonella* serovar Enteritidis (a serovar similar to Typhimurium) preincubated with AHL (C12) increased their persistence in the hemolymph with minor to no significant effects on host survival or health (Luiz de Freitas et al. 2021). It was not determined if this phenotype was *sdiA*-dependent. It is also unfortunate that C12 was used, as this AHL is not detected by SdiA (Smith and Ahmer 2003). Our lab has investigated the potential of *Salmonella* SdiA activation and fitness in *Drosophila melanogaster*, but no SdiA activity or *sdiA* phenotypes were observed (Ahmer, unpublished). We then performed similar experiments in house flies (*Musca domestica*) and observed sporadic activation that is worthy of further study (Ahmer, unpublished).

### Reptiles

Turtles are an asymptomatic carrier of nontyphoidal *Salmonella* and a source of outbreaks in the USA (Hidalgo-Vila et al. 2007, Back et al. 2016). A study in our lab found that SdiA activation occurs within the turtle intestine at levels comparable to those observed after growth *in vitro* in the presence of AHL (Smith et al. 2008). The source of AHLs was most likely the cocolonizing aquatic pathogen, *Aeromonas hydrophila*, a producer of AHLs detectable by *Salmonella* (Smith et al. 2008). Despite strong activation, *sdiA* mutants have no fitness defects in this system. The microbiota of turtles appears to be more abundant in Proteobacteria than that of humans, though their composition is impacted by many factors including location, age, and captivity status (reviewed in (Kuschke 2022)). Other *Salmonella* subspecies (that generally do not infect humans) are often isolated from turtles and encode *sdiA*, but *in vivo* studies are limited to *S*. Typhimurium (Hidalgo-Vila et al. 2007).

### Other

Experimental infections of guinea pigs, rabbits, pigs, and chickens (chicks) have been evaluated as a site of *Salmonella* SdiA activity (Smith et al. 2008). No activation occurred in any tested host. Mutant phenotypes of *sdiA* were found only in chicks, but the mutation was advantageous, and magnitude of the phenotype was small (<3-fold).

## What are the phenotypes of *sdiA* mutants?

Other than a role in virulence or colonization of hosts, *sdiA* has a small number of reported phenotypes with a significant amount of literature (cell division, drug resistance, and biofilm formation). Other phenotypes are either discussed in other sections or not included in this review. Reproducibility is a significant issue in this aspect of SdiA research as phenotypes have been described using *sdiA* mutants or using plasmid-based expression of *sdiA*. The latter method often produces phenotypes and regulatory changes that are not observed when *sdiA* is expressed from the chromosome under its native promoter. This disparity can be interpreted as artifacts arising from increasing the copy number of the gene in question, or it is possible that the observed behaviors require environmental conditions that are currently unknown. Studies relying on *sdiA* mutants sometimes produce phenotypes and regulatory changes not observed in independent constructs, other strains/species, or occur only in AHL-independent manners. Thus, discerning behaviors relevant to the eavesdropping paradigm requires a more nuanced consideration of the data underlying each purported phenotype.

### Cell division

SdiA was initially discovered in an early study on nearby gene *uvrC* in *E. coli* (Shanna et al. 1986). Shanna et al. described it as a “28 kDa protein” with a LexA-binding site in its terminator region and its higher rate of rare codons suggested a regulatory protein as proposed by Konigsberg and Godson (1983). After its initial description, SdiA was identified in a screen for genes involved in cell division performed by Wang et al. (1991). Specifically, they selected for clones in a plasmid-based *E. coli* DNA library that could rescue growth upon overexpression of MinCD, which inhibits assembly of the Z-ring and thus septation and cell division. They observed that two genes, *ftsZ* and the 28 kDa protein, could suppress division inhibition (hence SdiA). Based on their findings that (i) overexpressing *sdiA* produced mini-cells, (ii) *sdiA* mutants had no cell division phenotypes, and (iii) *sdiA* could not complement *ftsZ* mutants, they surmised that *sdiA* was a positive regulator of the *ftsQAZ* locus (Wang et al. 1991).

A follow-up study described two promoters of *ftsQAZ*: one regulated by RpoS and one by SdiA (Sitnikov et al. 1996). The SdiA regulated promoter (P2) could indeed be activated by overexpression of SdiA and activity increased 2-fold by introduction of exogenous AHLs. The P2 promoter has also been shown to be bound by SdiA directly using gel-shift assays (Kanamaru et al. 2000, Yamamoto et al. 2001, Kim et al. 2014). The mini-cell phenotype resulting from SdiA interaction with the *ftsQAZ* promoter has been observed by multiple researchers (Wang et al. 1991, Ahmer et al. 1998, Kanamaru et al. 2000). The major caveat to this finding is that the cell division phenotype, as well as the regulation of the P2 promoter, has only been observed using plasmid-based expression of SdiA. When examining a native expression system (i.e. wild-type *E. coli*), the introduction of AHLs has no effect on cell division or *ftsQAZ* expression, and an *sdiA* mutation has no effect on cell division or *ftsQ* promoter regulation (Wang et al. 1991, Dyszel et al. 2010b). However, an *sdiA* mutant of *Klebsiella* was recently reported to have a filamentation phenotype that could be rescued by plasmid complementation (Pacheco et al. 2021). Although bacteria can feed information about stress and metabolic state into cell division machinery, this often occurs at the posttranscriptional level (Chen et al. 2012, Hill et al. 2013). It is not clear why the presence of foreign AHL producers would be linked to cell division. The regulation of cell division may be an artifact of plasmid-based expression of *sdiA*, or it is possible that SdiA regulates cell division in a specific environmental condition yet to be discovered.

### Multiple-drug resistance

The multiple-drug resistance phenotype of SdiA was implicated in a microarray study by Wei et al. (2001) comparing *E. coli* overexpressing SdiA from a plasmid to a vector control. The AcrAB system, a TolC-dependent efflux pump which confers resistance to multiple compounds, was upregulated compared to a vector control (Wei et al. 2001, Pos 2009). Overexpression of SdiA in *E. coli* and *Cronobacter* has also been shown to increase resistance to several antibiotics (Rahmati et al. 2002, Dyszel et al. 2010b, Tavio et al. 2012, 2014). Mutation of *sdiA* alone has little effect on drug resistance in *E. coli* (Rahmati et al. 2002, Dyszel et al. 2010b, Hai et al. 2024), *Salmonella* (Dyszel et al. 2010b), or *Cronobacter* (Cheng et al. 2022) and AHLs have no effect on resistance in *E. coli* or *Salmonella* (Dyszel et al. 2010b, Schwieters and Ahmer 2024). Therefore, while there is currently very little evidence that SdiA regulates drug resistance, it is possible that a yet undiscovered environmental condition is required to observe this regulation.

### Biofilms

The relationship between SdiA and biofilms was first suggested in two studies from Lee et al. (2007, 2008). Using microarrays and mutant studies in *E. coli*, there were four observations: (1) *sdiA* mutants have increased biofilm formation, (2) *sdiA* mutants differentially express curli and flagella genes, (3) biofilm formation can be suppressed by indole in a *sdiA-*dependent manner, and (4) this occurs primarily at lower temperatures (30°C) (Lee et al. 2007, 2008). A later study evaluated the role of SdiA, AHLs, and indole in biofilms of both *E. coli* and *Salmonella* (Sabag-Daigle et al. 2012). For *E. coli*, mutation of *sdiA* has no effect on biofilm formation at any temperature (25°C, 30°C, and 37°C) and indole suppresses biofilm formation in *E. coli*, but in a *sdiA-*independent manner. Other studies suggest mutating *sdiA* in pathogenic *E. coli* strains can alter biofilm formation (Culler et al. 2018, Hai et al. 2024). For *Salmonella*, neither *sdiA* nor indole has any effect on biofilms (Sabag-Daigle et al. 2012). Interestingly, indole can prevent AHL-dependent activation of SdiA regulated genes (Sabag-Daigle et al. 2012), which may have implications for eavesdropping in the human gut where indole is at relevant concentrations (Karlin et al. 1985, Zuccato et al. 1993). Biofilm phenotypes have also been reported in *sdiA* mutants of *Cronobacter* (Cao et al. 2022, Cheng et al. 2022), *Enterobacter* (Shankar et al. 2013), and *Klebsiella* (Pacheco et al. 2021) but no study has reported significant AHL-dependent changes in biofilm formation. Interestingly, motility is implicated in both *E. coli* and *Salmonella* biofilms (Wood et al. 2006, Simm et al. 2014). Mutation of *sdiA* in *E. coli*, but not *Salmonella*, causes motility defects (Dyszel et al. 2010b). The *sdiA* gene itself is regulated by FliA in *Salmonella*  ^76^. Thus, SdiA is within regulatory networks that are involved with biofilm formation and more investigation is warranted.

## What genes does SdiA regulate?

As SdiA is a transcription factor, perhaps the most important question is “what does it regulate?”. Possible regulon members have been identified with microarrays (Wei et al. 2001, Lee et al. 2008), genetic screens (Ahmer et al. 1998, Van Houdt et al. 2006, Dyszel et al. 2010b, Sabag-Daigle et al. 2015), and RNA-seq (Cheng et al. 2022, Schwieters and Ahmer 2024). These studies compare wild-type to mutant (Lee et al. 2008, Cheng et al. 2022, Schwieters and Ahmer 2024) or use plasmid overexpression to induce activity (Ahmer et al. 1998, Wei et al. 2001, Schwieters and Ahmer 2024). Some but not all have used AHLs as part of their initial screen, either with (Schwieters and Ahmer 2024) or without (Van Houdt et al. 2006, Dyszel et al. 2010b, Sabag-Daigle et al. 2015) a *sdiA* mutant control. Screens often identified dozens or hundreds of putative *sdiA* regulated genes, but viewed stringently, the size of the regulons may be much smaller (<20 genes). Several previously uncharacterized genes have been renamed to *srg* (SdiA regulated genes). The regulon of each genus is described below.

### Salmonella

Two studies have attempted to identify the SdiA regulon of *Salmonella*, one with a genetic screen (Ahmer et al. 1998) and one with RNA-seq (Schwieters and Ahmer 2024). A major limitation in studying *Salmonella* SdiA is its direct regulation by FliA (Plitnick et al. 2020). Activity is strongest in motility agar (Smith and Ahmer 2003) and we have been unable to extract RNA from semisolid media suitable for downstream sequencing. As an alternative approach, we identified putative hits by expressing *sdiA* on a plasmid. Of the many putative hits, six loci encoding 18 genes have been “validated” in that they are activated or repressed by *sdiA* expressed from the chromosome, and the effects may or may not require the presence of AHLs (Schwieters and Ahmer 2024). It should be noted that only the *pefI-srgC* operon is known to be directly regulated by SdiA (Abed et al. 2014). All other loci described below may be directly or indirectly affected by SdiA.

The *S*. Typhimurium SdiA regulon includes the *pefI-srgC* operon, *srgE, srgF, srgGH, srgKJ*, and *menFDHBCE* (Schwieters and Ahmer 2024). The six gene operon encoding *pefI, srgD, srgA, srgB, rck*, and *srgC* is encoded on the virulence plasmid pSLT (reviewed in Mambu et al. 2017). Our understanding of this operon is still limited. SrgB, a putative lipoprotein, and SrgC, a transcriptional regulator, are yet to be characterized. PefI and SrgA are involved in expression of Pef fimbriae through their roles as a transcriptional regulator and in posttranslational maturation of PefA, respectively (Bouwman et al. 2003). Two studies have suggested a role for PefI and/or SrgD in regulation of flagellar motility (Wozniak et al. 2009, Wallar et al. 2011). Although motility, through FliA, is an essential regulator of SdiA (Plitnick et al. 2020), neither mutation of *sdiA* nor AHLs have any effect on either transcription of motility genes, or motility phenotypes (Smith and Ahmer 2003, Schwieters and Ahmer 2024). The best characterized among these six genes is *rck*, which encodes an outer membrane protein that confers resistance to complement killing and mediates invasion of host cells (Cirillo et al. 1996, Rosselin et al. 2010). Rck is only weakly expressed during infection of mice (Koczerka et al. 2024) and binds epidermal growth factor receptor (Wiedemann et al. 2016). The second *S*. Typhimurium specific *sdiA* regulated gene is *srgE*, which encodes a secreted effector (Habyarimana et al. 2014). *Salmonella* encodes two separate type three secretion systems, one involved in invasion (SPI1) and one involved with intracellular survival and replication (SPI2) (Fàbrega and Vila 2013). SrgE is secreted in a SPI2-dependent manner, suggesting a role in intracellular pathogenesis (Habyarimana et al. 2014).

The remaining four loci of the *S*. Typhimurium *sdiA* regulon, *srgF, srgKJ, srgGH*, and *menFDHBCAE*, have not been well characterized (Schwieters and Ahmer 2024). SrgF is a putative ATP-dependent RNA helicase-like protein, though bioinformatic tools find no similarity to known protein domains (Zimmermann et al. 2018, van Kempen et al. 2023). SrgF has a high degree of basal expression in *S*. Typhimurium, especially compared to *sdiA* and other regulon members (Kröger et al. 2013). It appears sparsely in the literature, with putative mutant phenotypes in chicken colonization, motility, and phage defense (Wang et al. 2006, Haznedaroglu et al. 2009, Knudsen et al. 2012, Chaudhuri et al. 2013, Adler et al. 2021). No *srgF* fitness defects in mice were found (Schwieters and Ahmer 2024). SrgKJ are a band 7/mec-2 family protein and NfeD family protein, respectively (Chiba et al. 2006). A study of *E. coli*’s SrgK ortholog QmcA found that it could rescue lethal mutations in proteases, indicating a role in protein turnover with YbbJ acting as a helper protein (*qmcA* is not *sdiA* regulated in *E. coli*) (Chiba et al. 2006, Schwieters and Ahmer 2024). Orthologs of SrgKJ are conserved in Gram-negative bacteria and *sdiA* regulation of *srgKJ* orthologs occurs in both *E. cloacae* and *Salmonella* (Schwieters and Ahmer 2024). The protein target(s) of SrgKJ remain unidentified and its connection to eavesdropping is unclear. SrgGH are both truncated in *S*. Typhimurium. SrgG encodes the N-terminus of a full-length citrate transporter found in *S. bongori* and *E. cloacae*, and *E. cloacae srgG* is also *sdiA* regulated (Schwieters and Ahmer 2024). SrgH, like SrgG, is a fragment of a nearby protein, UshB (Cdh in *E. coli*). It is unknown if SrgG or SrgH are made and if these truncated proteins perform any relevant functions in *Salmonella*. The last *sdiA* regulated locus is the *menFDHBCAE* operon, which produces menaquinones (vitamin K2) that are involved in electron transport (Dahm et al. 1998, Buss et al. 2001).

Serovar Typhimurium can be found in numerous human food related environments and colonizes a wide range of hosts including humans, livestock, plants, reptiles, and insects (Pedersen et al. 2009, Wales et al. 2010, Schikora et al. 2012). The possible sites where SdiA could be used to eavesdrop on foreign bacteria is vast. At the same time, a recently emerged (∼50 000 years ago) serovar of *Salmonella*, Typhi, is believed to use humans as its sole host and reservoir (Kidgell et al. 2002). Their limited niche overlap, we hypothesized, represents a selective pressure on their *sdiA* regulons. After elucidating the regulons of both Typhimurium and Typhi, we found their regulons to partially overlap: four loci regulated in both serovars (*srgF, srgKJ, srgGH*, and *menFDHBCE*), two specific to Typhimurium (*pefI-srgC* and *srgE*), and one specific to Typhi (*srgIL*) (Schwieters and Ahmer 2024). Typhi does not harbor pSLT and instead encodes a subset of the *pefI-srgC* operon on its chromosome that is not *sdiA* regulated (Koczerka et al. 2021, Schwieters and Ahmer 2024). The sole regulon member specific to *S*. Typhi, *srgIL*, encodes two small lipoproteins orthologous to *yfgHI* in *E. coli* (Schwieters and Ahmer 2024). Based on the reported sensitivity of *E. coli yfgI* mutants to nalidixic acid (Skunca et al. 2013), we examined a possible relationship between eavesdropping and resistance to DNA damage. Surprisingly, neither serovar Typhimurium nor Typhi exhibit any *sdiA* or AHL-dependent changes in resistance to nalidixic acid or UV damage (Schwieters and Ahmer 2024).

### Escherichia

Of the many studies on *E. coli sdiA*, the glutamate-dependent acid fitness island (*gad*, reviewed in Kanjee and Houry 2013) and Locus of Enterocyte Effacement (LEE) are the two best described regulon members of *E. coli*. Regulation of *gad* occurs in both nonpathogenic (K12) and pathogenic (O157:H7) strains and has been described by multiple labs (Hughes et al. 2010, Dyszel et al. 2010b, Ma et al. 2020). A significant amount of *sdiA*-dependent regulation of *gad* is AHL-independent (Hughes et al. 2010, Dyszel et al. 2010b) and acid resistance phenotypes are stronger at lower temperatures, at least in K12 (Van Houdt et al. 2006, Dyszel et al. 2010b). Regulation of LEE by SdiA occurs directly at the promoter of virulence regulator *ler*, with stronger AHL-dependent regulatory phenotypes than *gad* despite the presence of both AHL-dependent and -independent binding sites on the promoter (Kanamaru et al. 2000, Hughes et al. 2010, Nguyen et al. 2015). SdiA also represses flagellar genes in *E. coli*, with *sdiA* mutants reported to have motility defects (Wei et al. 2001, Sharma et al. 2010, Dyszel et al. 2010b). A few other regulon members have been reported including the transcription factor *uvrY* (which is near *sdiA*) (Suzuki et al. 2002, Van Houdt et al. 2006), ribosome modulation factor *rmf* (Yoshida et al. 2018), and an O-antigen chain length determinant *fepE* (Schwieters and Ahmer 2024). Although the regulatory mechanism is unknown, it was found that AHLs can induce temperate phages in *E. coli* in a *sdiA-*dependent manner (Ghosh et al. 2009).

### Enterobacter

The SdiA regulon of *Enterobacter cloacae* includes a handful of *Enterobacter* specific genes encoding hypothetical proteins of unknown function. The regulon also includes *srgKJ* (described above), the copper transporter *copA*, the O-antigen chain length determinant *fepE*, signal transduction proteins, components of a putative type 6 secretion system, a phage integrase, the menaquinone biosynthesis operon, and a full length version of citrate transporter *srgG* (Sabag-Daigle et al. 2015, Schwieters and Ahmer 2024). The regulatory action of SdiA in *E. cloacae* is more complex: a mix of activation and repression occurring in both AHL-dependent and -independent manners. As we found in *Salmonella*, this regulon is difficult to understand as these genes do not appear to be related in function and most have no reported phenotypes. Direct regulation by SdiA has not been demonstrated for any locus in *E. cloacae*.

### Other eavesdropping genera

Of the other genera encoding *sdiA, Klebsiella* has one published study (Pacheco et al. 2021) and *Cronobacter* has two (Cao et al. 2022, Cheng et al. 2022). It was reported that *sdiA* mutation in *Klebsiella pneumoniae* alters the expression of *rpoS* and *ftsQ* (<2-fold). Gel-shift assays support SdiA binding to both *ftsQ* and *fimA* promoters in *K. pneumoniae*, but it was not determined if regulation was AHL-dependent (Pacheco et al. 2021). Additionally, this mutant has increased expression of type 1 fimbriae, which is also regulated by phase variation (Abraham et al. 1985, Clegg et al. 1996, Kolenda et al. 2019). It is unclear if *sdiA* regulates phase variation. In our most recent study, we tested whether *S*. Typhimurium *sdiA* regulated type 1 fimbriae (which is controlled by a different phase variation mechanism) (Kolenda et al. 2019, Schwieters and Ahmer 2024). In *S*. Typhimurium, plasmid-based expression of *sdiA* could repress expression of the four promoters encoding the structural genes and three regulators (*fimW, fimY*, and *fimZ*), but there was no regulation under endogenous expression conditions (Schwieters and Ahmer 2024) or evidence that *sdiA* controls phase variation (unpublished data).

*Cronobacter sakazakii* has been examined with an RNA-seq experiment comparing wild-type to *sdiA* mutant without the addition of AHLs (Cao et al. 2022, Cheng et al. 2022). The results suggest that SdiA represses flagellar genes and activates biofilm component genes (cellulose and extracellular polysaccharide). Mutant phenotypes were consistent with those changes, but it was not determined if motility or biofilm formation phenotypes were AHL-dependent (Cao et al. 2022).

## Conclusions and future directions in the field of SdiA-mediated eavesdropping

Several genera within the Enterobacteriaceae encode SdiA, a LuxR-type protein. By loss of the corresponding AHL synthase, these bacteria no longer use AHLs to facilitate population-density-dependent behaviors (quorum sensing) but instead detect the AHLs produced by other bacterial species in their environment (eavesdropping). Here, we have discussed three questions fundamental to the nature of SdiA: How does SdiA regulate genes, where is SdiA relevant, and what does SdiA regulate? By finding the answers to these questions, we may understand this simple yet nebulous behavior in bacteria.

Many studies have probed various hosts as a relevant site of SdiA activation, but significant mutant defects, a strong indicator of biological relevance, are lacking. The most clinically relevant environment, the human gut, cannot be investigated directly. Our metagenomic search suggests that *luxI* homologs are infrequently detected in human gut metagenomes (Table 1) and mammalian infection models support the notion that they are unlikely to be a relevant environment for SdiA-mediated eavesdropping. This possibility should not be discounted outright as *sdiA* mutants do suffer fitness defects if *Salmonella* is engineered to produce its own AHLs. Additionally, SdiA regulons do contain putative virulence factors (e.g. *srgE*) (Dyszel et al. 2010a, Habyarimana et al. 2014). Environments known to include eavesdropping bacteria like *Salmonella* and *E. coli* and AHL-producing bacteria within the genera *Erwinia* and *Pantoea* (insects and plants) showed a higher frequency of detection for *luxI* homologs in our pilot metagenome search (Starr and Chatterjee 1972, Wales et al. 2010, Blazar et al. 2011, Schikora et al. 2012, Walterson and Stavrinides 2015). There are very few studies in these environments and further investigation into their potential is clearly warranted.

A significant number of studies on the SdiA regulon used plasmid-based expression for identification of regulon members and *in vitro* phenotypes. As discussed above, expressing *sdiA* on a plasmid yields more putative regulon members than *sdiA* expressed from its native position in the chromosome. It is not known if the genes that are regulated only in response to plasmid-encoded *sdiA* are artifacts or if they are true members of the *sdiA* regulon for which proper environmental conditions have not yet been found. SdiA’s ability to regulate in both ligand-dependent and -independent manners also complicates our understanding of what constitutes eavesdropping behavior (i.e. what genes are expressed in response to foreign AHLs). Identification of a relevant environment will likely significantly advance our understanding of this response as we can then modify our laboratory conditions to better mimic such an environment (e.g. temperature, nutrient availability, stressors, and so on). The proper environment may also provide new insight into the functions of SdiA regulated genes, many of which are uncharacterized.

The conservation of *sdiA* orthologs from genera ranging from *Erwinia* to *Salmonella* suggests that the eavesdropping behavior was likely acquired and subsequently maintained in excess of 100 million years (Ochman and Wilson 1987, Ochman and Groisman 1994, Lawrence and Ochman 1998). This time frame provided ample opportunity for niche diversification among the Enterobacteriaceae, such that loss of *sdiA* would be expected in certain lineages. Yet, this did not occur and the presence of *sdiA* is conserved. We interpret this to mean that evolving lineages have remained in AHL-laden environments, where eavesdropping is advantageous. Has this always been the same environment, did they spread to unique environments, or both? Some of the difficulty in understanding SdiA-mediated eavesdropping is highlighted in the recently emerged lineage of *S*. Typhi, where SdiA is still responsive to AHLs and even has a strain specific regulon (Schwieters and Ahmer 2024). *S*. Typhi is limited to humans as a sole host and reservoir and humans do not appear to be a relevant site of SdiA-mediated eavesdropping. Is it possible that *S*. Typhi has a secondary reservoir, where SdiA is relevant, or is SdiA relevant in humans?

An apparent paradox in the SdiA-mediated eavesdropping paradigm is the conservation of foreign AHL detection and the divergent transcriptional responses among species. If eavesdropping occurs in a common environment, why are the regulons so different? Only a few regulon members have been found to overlap between genera and no gene is known to be *sdiA* regulated in more than two genera (Schwieters and Ahmer 2024). The absence of a “core” SdiA regulon suggests each genus or species responds to this external signal differently, but our understanding of SdiA regulons is far from complete and may suffer from study under nonideal conditions. SdiA may have a core regulon common to all genera, and auxiliary regulons that become more specific down the phylogenetic tree (e.g. species specific and strain specific).

Alternatively, a core regulon may not exist, but rather SdiA-mediated eavesdropping could lead to a common phenotypic response mediated by different regulated genes. In this review of the literature, we noticed a reoccurring theme of response to phage. In *E. coli*, AHLs can induce temperate phage induction (Ghosh et al. 2009). In *Salmonella*, plasmid expression of *sdiA* represses a significant number of prophage genes and some regulon members are implicated in phage defense (Adler et al. 2021, Schwieters and Ahmer 2024). A phage integrase is regulated by *sdiA* in *E. cloacae* (Sabag-Daigle et al. 2015) and O-antigen chain length determinant *fepE* is *sdiA* regulated in both *E. coli* and *E. cloacae*, which could influence phage adsorption (Schwieters and Ahmer 2024). The possibility of SdiA-mediated eavesdropping as a regulator of phage defense is worthy of more investigation.
